# Pharmacokinetics and Pharmacodynamics of Clofazimine for Treatment of Cryptosporidiosis

**DOI:** 10.1128/AAC.01560-21

**Published:** 2022-01-18

**Authors:** Cindy X. Zhang, Melissa S. Love, Case W. McNamara, Victor Chi, Ashley K. Woods, Sean Joseph, Deborah A. Schaefer, Dana P. Betzer, Michael W. Riggs, Pui-Ying Iroh Tam, Wesley C. Van Voorhis, Samuel L. M. Arnold

**Affiliations:** a Department of Pharmaceutics, University of Washington, Seattle, Washington, USA; b Calibr, The Scripps Research Institute, La Jolla, California, USA; c School of Animal and Comparative Biomedical Sciences, College of Agriculture and Life Sciences, University of Arizona, Tucson, Arizona, USA; d Malawi-Liverpool Wellcome Trust Clinical Research Programme, Blantyre, Malawi; e Liverpool School of Tropical Medicine, Liverpool, United Kingdom; f Department of Medicine, University of Washington, Seattle, Washington, USA

**Keywords:** cryptosporidiosis, pharmacokinetics, pharmacodynamics, PK/PD, gastrointestinal, infectious diseases, *Cryptosporidium*, gastrointestinal infection

## Abstract

Infection with *Cryptosporidium* spp. can cause severe diarrhea, leading to long-term adverse impacts and even death in malnourished children and immunocompromised patients. The only FDA-approved drug for treating cryptosporidiosis, nitazoxanide, has limited efficacy in the populations impacted the most by the diarrheal disease, and safe, effective treatment options are urgently needed. Initially identified by a large-scale phenotypic screening campaign, the antimycobacterial therapeutic clofazimine demonstrated great promise in both *in vitro* and *in vivo* preclinical models of *Cryptosporidium* infection. Unfortunately, a phase 2a clinical trial in HIV-infected adults with cryptosporidiosis did not identify any clofazimine treatment effect on *Cryptosporidium* infection burden or clinical outcomes. To explore whether clofazimine’s lack of efficacy in the phase 2a trial may have been due to subtherapeutic clofazimine concentrations, a pharmacokinetic/pharmacodynamic modeling approach was undertaken to determine the relationship between clofazimine *in vivo* concentrations and treatment effects in multiple preclinical infection models. Exposure-response relationships were characterized using *E*_max_ and logistic models, which allowed predictions of efficacious clofazimine concentrations for the control and reduction of disease burden. After establishing exposure-response relationships for clofazimine treatment of *Cryptosporidium* infection in our preclinical model studies, it was unmistakable that the clofazimine levels observed in the phase 2a study participants were well below concentrations associated with anti-*Cryptosporidium* efficacy. Thus, despite a dosing regimen above the highest doses recommended for mycobacterial therapy, it is very likely the lack of treatment effect in the phase 2a trial was at least partially due to clofazimine concentrations below those required for efficacy against cryptosporidiosis. It is unlikely that clofazimine will provide a remedy for the large number of cryptosporidiosis patients currently without a viable treatment option unless alternative, safe clofazimine formulations with improved oral absorption are developed. (This study has been registered in ClinicalTrials.gov under identifier NCT03341767.)

## INTRODUCTION

The symptoms of cryptosporidiosis, the disease caused by parasitic infections with *Cryptosporidium* spp., include prolonged episodes of watery diarrhea along with other symptoms, such as stomach pain, dehydration, nausea, vomiting, and fever. While cryptosporidiosis is most commonly self-limiting in healthy adults, the disease can cause recurrent or chronic diarrhea in children and immunocompromised individuals and can lead to death in severe cases (1, 2). Cryptosporidiosis is also highly associated with long-term adverse impacts, including growth stunting, poor physical fitness, and poor cognitive development when infections occur at a young age, even when the infections are asymptomatic (3–7). A reanalysis of the Global Enteric Multicenter Study (GEMS) revealed that *Cryptosporidium* was one of the top six pathogens responsible for moderate to severe diarrhea in children younger than 5 years in Africa and Asia (8). For children under the age of 1 year, *Cryptosporidium* was the third most common cause of moderate to severe diarrhea, ranking higher than *Shigella*, norovirus, and Salmonella. Due to underdiagnosis and underappreciation of the disease, the true global burden of cryptosporidiosis is not known. The pervasive impact of cryptosporidiosis was illustrated by The Global Burden of Disease 2016 study, which estimated that over 44.8 million episodes of diarrhea and 48,000 deaths globally can be attributed to cryptosporidiosis annually (9). *Cryptosporidium* infections, while exerting the most detrimental effects in underresourced countries, are also common in high-income countries. Due to the parasite’s highly infectious nature and its resistance to water chlorination, it is a leading cause of waterborne disease in the United States (10–15).

As a historically neglected disease, cryptosporidiosis has only one drug, nitazoxanide, approved for treatment by the U.S. Food and Drug Administration (FDA). While nitazoxanide is effective in treating otherwise healthy adults, with parasite clearance reported in up to 93% of treated subjects (compared to 37% with placebo treatment) (16), its efficacy is greatly compromised in malnourished children, in whom the response rate dropped to only 56% following nitazoxanide treatment, compared to 23% with placebo control (17). No significant treatment benefit was observed when nitazoxanide was compared to placebo in Zambian children living with HIV (17, 18), supporting the belief that the efficacy of nitazoxanide is heavily dependent on the competency of infected individuals’ immune systems. Unfortunately, immunocompromised individuals and malnourished children, who may not benefit from nitazoxanide treatment, are those who are most vulnerable and suffer the most severe consequences of *Cryptosporidium* infection. Therefore, there is an urgent need for an effective and safe treatment that can be used to treat malnourished children and the immunocompromised. A traditional approach for drug discovery in cryptosporidiosis would require screening against large collections of small molecules, and any hits would most likely need extensive optimization to improve safety, pharmacokinetics, and/or potency. While there are many promising, novel treatments in development, it is not clear if/when any of these new therapeutic candidates will be available in the clinic. Drug repurposing provides an alternative approach to cryptosporidiosis drug development, and repurposing candidates will often already have established safety, toxicity, and pharmacokinetic data (19, 20). Thus, drug repurposing can facilitate the delivery of new therapeutic interventions to the clinic by reducing the time and resources required for development.

Through an unprecedented phenotypic screening campaign by the California Institute for Biomedical Research (Calibr), ∼80,000 compounds were screened *in vitro* against *Cryptosporidium*, and it was observed that the antimycobacterial clofazimine was active against both Cryptosporidium parvum and Cryptosporidium hominis (21). Clofazimine is an FDA-approved antimicrobial agent for the treatment of lepromatous leprosy (22, 23), and the therapeutic demonstrated *in vivo* efficacy in a preclinical mouse model of *Cryptosporidium* infection (21). Nevertheless, when a phase 2a clinical trial conducted in HIV-infected adults investigated clofazimine as a potential treatment of cryptosporidiosis, clofazimine treatment did not result in improved microbiological or clinical outcomes compared to placebo (24). The lack of treatment effect was observed with a clofazimine dosing regimen exceeding the daily recommended dosage and was the maximum given in clinical practice. In an effort to determine whether higher clofazimine concentrations in the phase 2a trial may have led to a favorable therapeutic response, we undertook a pharmacokinetic/pharmacodynamic (PK/PD) modeling approach informed by previously unpublished preclinical *in vivo* studies to develop exposure-response relationships for clofazimine treatment of *Cryptosporidium* infection.

## RESULTS

### Clofazimine pharmacokinetics and efficacy in mice.

When administered orally to mice as single doses ranging from 0.03 mg/kg of body weight to 300 mg/kg, clofazimine was observed to have nonlinear kinetics (Table 1). While the reason for the nonlinearity is unclear, the less-than-proportional increase in exposure with increasing dose may reflect a reduction in gastrointestinal absorption (i.e., lower oral bioavailability) due to its poor aqueous solubility.

**TABLE 1:** Clofazimine pharmacokinetics in mice after a single oral dose^*a*^

Dose (mg/kg)	*t*_1/2_ (h)	*C*_max_ (ng/mL)	*T*_max_ (h)	AUC (h·ng/mL)			CL/*F* (mL/min/kg)
				0–24 h	0–240 h	0–infinity	
0.03	ND	10	3	174	465	ND	0.9
0.1	70	22	8	401	1,446	1,580	1.1
0.3	86	52	1	973	3,781	4,465	1.1
1	87	99	8	2,082	10,123	11,887	1.4
3	106	168	3	3,265	16,841	20,794	2.4
10	120	342	8	7,420	38,771	53,161	3.1
30	106	626	8	14,053	74,933	96,592	5.2
100	183	1,550	3	26,997	139,253	228,440	7.3
300	118	2,153	3	44,914	248,866	338,831	14.8

a ND, values were not provided because only two time points were available to calculate *t*_1/2_ and AUC_∞_ for the 0.03-mg/kg dose group. *t*_1/2_, half-life; *C*_max_, maximum concentration of drug in plasma concentration; *T*_max_, time to *C*_max_; CL, clearance.

**FIG 1:**
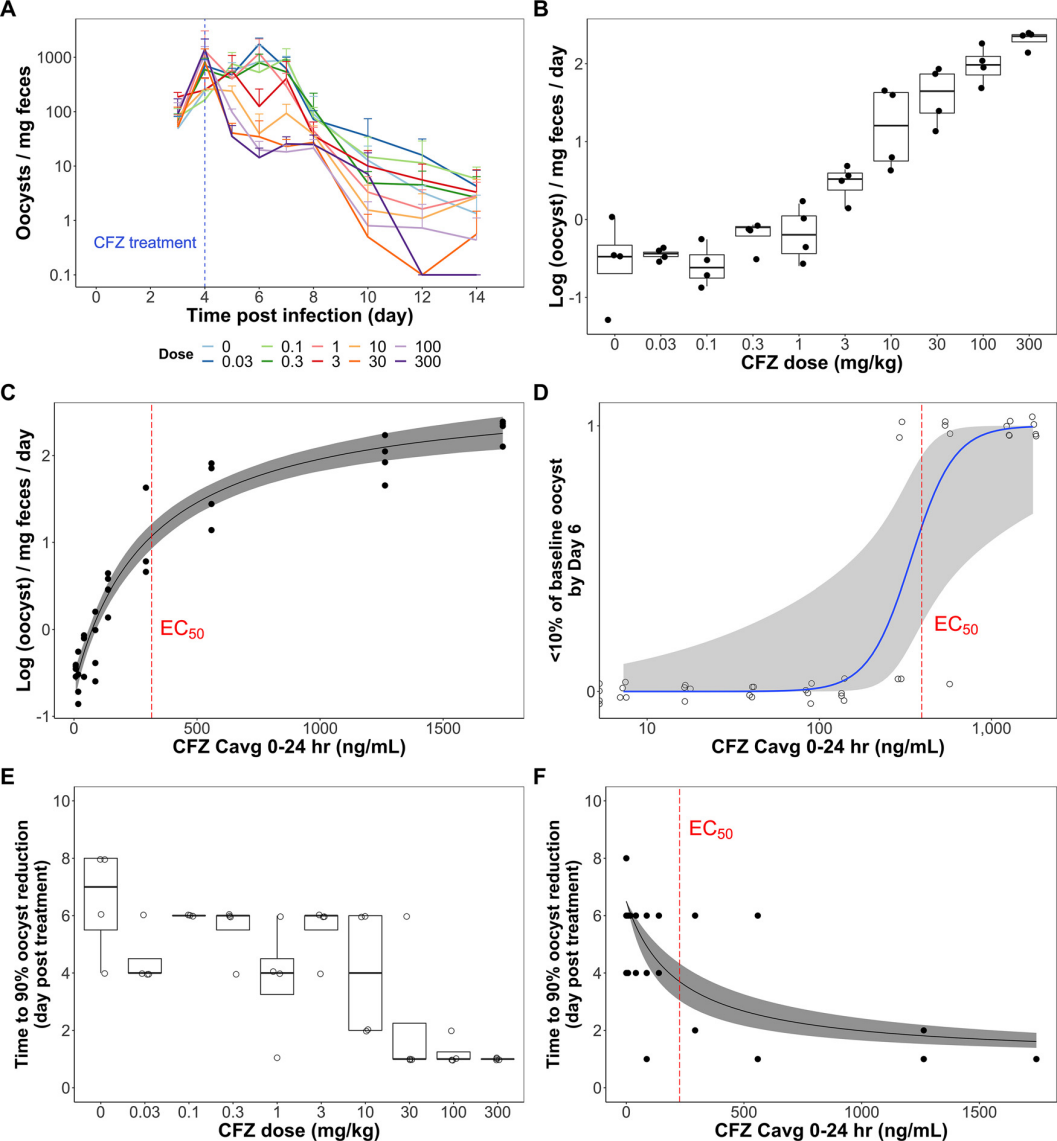
Clofazimine *in vivo* efficacy in mouse model of *Cryptosporidium* infection. (A) Mean oocyst shedding profile for each clofazimine treatment group plotted over time. A single clofazimine oral dose of various amounts was administered 4 days after oral inoculation with C. parvum. Each dose group contained four mice, and the fecal oocyst concentration for each mouse was quantified by flow cytometry. (B) Rate of reduction in log-transformed fecal oocyst concentration from day 4 to day 6 postinfection, summarized by dose group. The tops and bottoms of the boxes are the 75th and 25th percentiles, respectively, and the center line is the median. The whiskers indicate the range of data distribution that are within 1.5-fold of the interquartile range (IQR). Points that lie out of the reach of the whiskers are outliers by the 1.5 IQR rule. (C) A simple *E*_max_ model (black solid line) with 90% confidence interval (gray shaded area) was fitted to describe the relationship between average clofazimine concentration over the 24 h postdosing (*C*avg_0–24_) and the rate of oocyst reduction, with the model-predicted EC_50_ marked by the red dashed line. A dichotomous approach was also taken, with an event (“1”) being defined as having less than 10% of baseline oocyst load on day 6 postinfection. (D) Logistic regression model (blue solid line) with 90% confidence interval (gray shaded area), with the model predicted EC_50_ indicated by the red dashed line. (E and F) Last, a time-to-event outcome variable approach was employed. The number of days each mouse took to reach a 90% reduction in oocyst burden compared to the baseline oocyst concentration is summarized by dose group (E) or compared to *C*avg_0–24_ (F). The box plots in panel E were generated in the same fashion as those in panel B. A simple *E*_max_ model (black solid line) with 90% confidence interval (gray shaded area) was built to describe the correlation between the number of days and clofazimine *C*avg_0–24_ (F). The red dashed line represents EC_50_.

The efficacy of clofazimine in a mouse model of *Cryptosporidium* infection was dose dependent. In the mouse efficacy study, a single, oral dose of clofazimine ranging from 0.03 mg/kg to 300 mg/kg was administered 4 days after oral inoculation with 5 × 10^6^ C. parvum oocysts/mouse (*n* = 4/group) and oocyst shedding was measured daily up to 10 days after treatment initiation. It was observed that while oocyst counts declined over time for all treatments, a dose-dependent difference in the rate and onset of decline existed over the first 2 days posttreatment (Fig. 1A). The results, when summarized by dose group, demonstrated that a reduction in oocyst shedding rate between day 4 and day 6 postinfection was absent for clofazimine doses lower than 3 mg/kg but started to manifest at clofazimine doses of ≥3 mg/kg (Fig. 1B). The reduction in oocyst shedding rate appeared to increase with increasing clofazimine dose and was the greatest at the highest dose, 300 mg/kg. When fitted with an *E*_max_ model (Fig. 1C; equation 1 [see Materials and Methods]), which describes the relationship between average clofazimine concentration over the 24 h posttreatment and the rate of oocyst reduction, it was estimated that E_0_ was −0.670 log(oocysts)/mg feces/day, *E*_max_ was 3.46 log(oocysts)/mg feces/day, the Hill coefficient was 1, and the 50% effective concentration (EC_50_) was 316 ng/ml with a 95% confidence interval of 205 to 480 ng/ml. These estimates were used to calculate an EC_90_ of 2,770 ng/ml with a 95% confidence interval of 1,840 to 4,320 ng/ml (i.e., an average concentration from 0 to 24 h [*C*avg_0–24_] associated with 90% of maximum reduction in oocyst shedding rate).

In addition to changes in oocyst shedding rate in the initial 2 days after clofazimine administration, a dichotomous variable was designed where “success” was defined as achieving a 90% reduction in oocyst counts by comparing day 6 oocyst shedding numbers to the baseline oocyst count on day 4 postinfection. A logistic model was developed to characterize the relationship between success and clofazimine *C*avg_0–24_ (Fig. 1D; equation 2). Based on this logistic model, the EC_50_, which is the concentration at which there is a 0.50 probability of achieving success, was estimated to be 394 ng/ml and the EC_90_ to be 589 ng/ml.

A time-to-event outcome variable was also used to identify differences in efficacy between clofazimine treatment groups. A success event was designated as a 90% reduction in oocyst infection burden by comparing oocyst shedding counts on each day to the baseline oocyst count at treatment initiation, similar to the dichotomous outcome approach. However, instead of focusing on whether success was achieved by a certain time point, the time-to-event variable evaluated how quickly success was achieved. At lower doses, success in oocyst shedding (i.e., success) was generally achieved around 6 days posttreatment (Fig. 1E). However, at higher doses of 30 mg/kg, 100 mg/kg, and 300 mg/kg, success was observed as early as 1 day posttreatment. The median number of days mice in these dose groups needed to achieve success was 1 day. The data were fitted with an *E*_max_ model to describe the relationship between time to success and *C*avg_0–24_ (Fig. 1F; equation 3). The curve illustrates that with increasing clofazimine concentrations, the time needed to achieve a 90% reduction in oocyst shedding was reduced. E_0_ was set to 6.50 days posttreatment, and *E*_max_ was fixed at −5.50 days posttreatment. The Hill coefficient was estimated to be 1, EC_50_ was 227 ng/ml, and EC_90_ was 2,040 ng/ml.

### Clofazimine pharmacokinetics and efficacy in calves.

**FIG 2:**
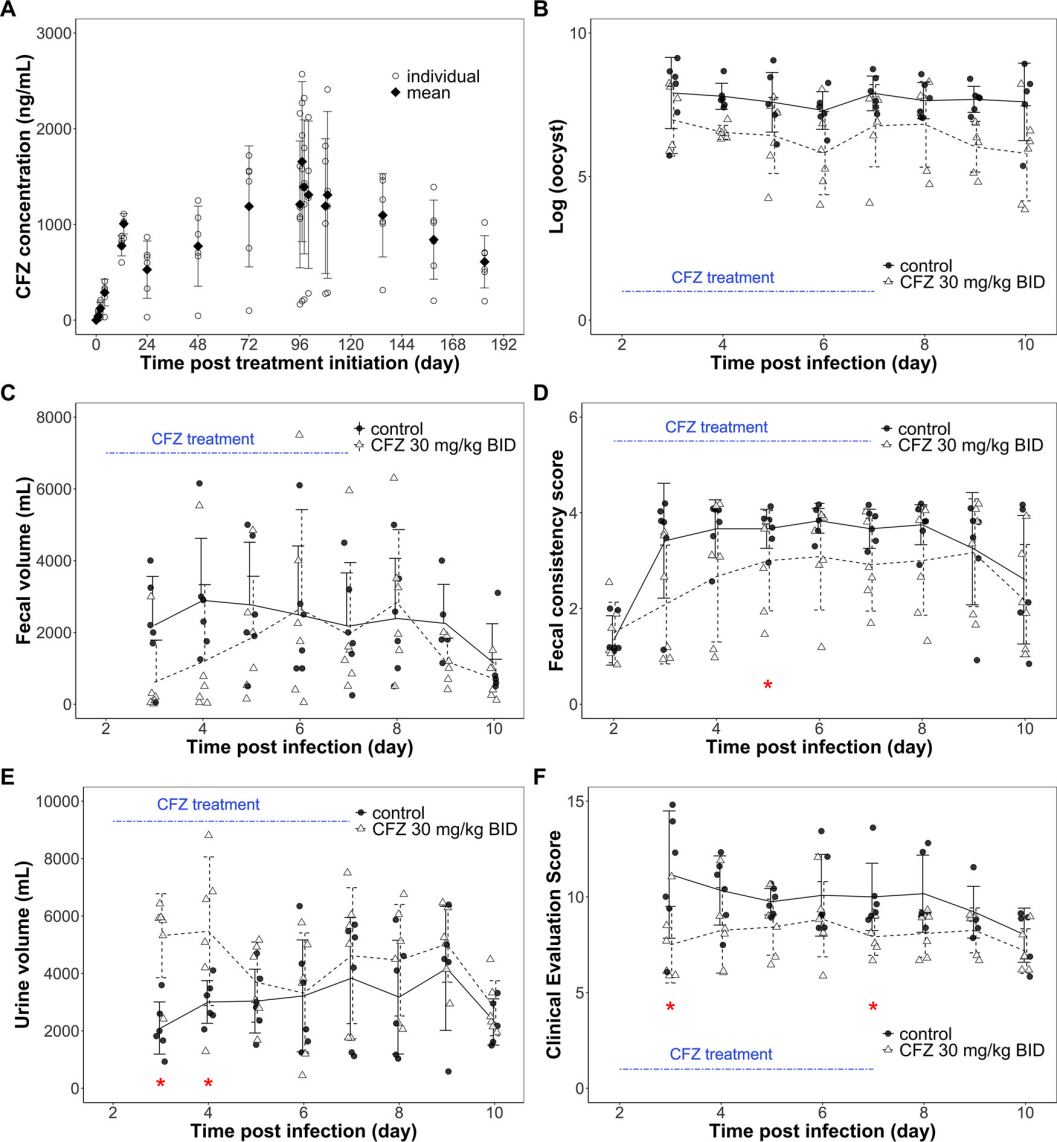
Clofazimine pharmacokinetics and pharmacodynamics in a calf model of cryptosporidiosis. Clofazimine was administered to calves with a dosing regimen of 30 mg/kg twice daily over 5 days for a total of 10 doses. (A) Up to 15 blood samples were collected from each calf (*n* = 6) over time, and mean and individual clofazimine concentrations were plotted. (B) Stool samples were collected every 24 h starting on day 3 postinfection for clofazimine- and vehicle control-treated calves, and fecal oocyst counts were quantified by real-time PCR. Mean and individual oocyst counts are plotted over time. (C to F) Other pharmacodynamic outcomes, including fecal volume (C), fecal consistency score (D), urine volume (E), and clinical evaluation score (F) were recorded daily and are shown as individual values and means (standard deviations are indicated by error bars). An asterisk indicates days on which the treatment group and the control group differed significantly (*P* ≤ 0.05) at the collection endpoint.

The microbiological and clinical efficacy of clofazimine treatment was characterized in an established calf model of cryptosporidiosis. Clofazimine concentrations observed with 30 mg/kg clofazimine twice a day (BID) for 5 days for the 6 calves in the treatment group are plotted in Fig. 2A. The mean ± standard deviation (SD) clofazimine concentrations over the initial 24 h (*C*avg_0–24_) and from 96 to 108 h after treatment initiation (*C*avg_96–108_) were 500 ± 248 ng/ml and 1,330 ± 605 ng/ml, respectively. Daily oocyst shedding counts for calves in the treatment and control groups demonstrated that the mean oocyst counts for the treatment group tended to be lower than those of the control group for the duration of the study (Fig. 2B), although the differences were not statistically significant with the single dosing regimen tested and the number of calves involved in this study. Clinical endpoints other than oocyst counts were also collected, including daily fecal volume, fecal consistency score, clinical evaluation score, and daily urine volume. The profiles of these endpoints in the treatment group and the control group animals are summarized in Fig. 2C to F. Daily fecal volume and fecal consistency scores are indicators of the severity of diarrhea. While the mean daily fecal volume profiles were not different between the control and the treatment groups, the mean fecal consistency score profiles suggested that the treatment group animals tended to have less severe diarrhea than the control group animals (Fig. 2C and D). The mean daily urine volume profile showed that the treatment group calves tended to produce more urine than the control group calves (Fig. 2E), with the difference being significant on days 3 and 4 postinfection (within 2 days after treatment initiation). Thus, there were fewer signs of dehydration in the treatment calves. The clinical evaluation score summarizes clinical symptoms, general health observations, presence or absence of diarrhea, and fecal consistency, where a lower score represents better clinical outcome. The mean clinical evaluation score profile for the two groups suggested that the treatment group tended to have lower clinical scores and thus better clinical outcomes (Fig. 2F). The clinical scores were significantly better for the treatment group than the control group on days 3 and 7 postinfection.

**FIG 3:**
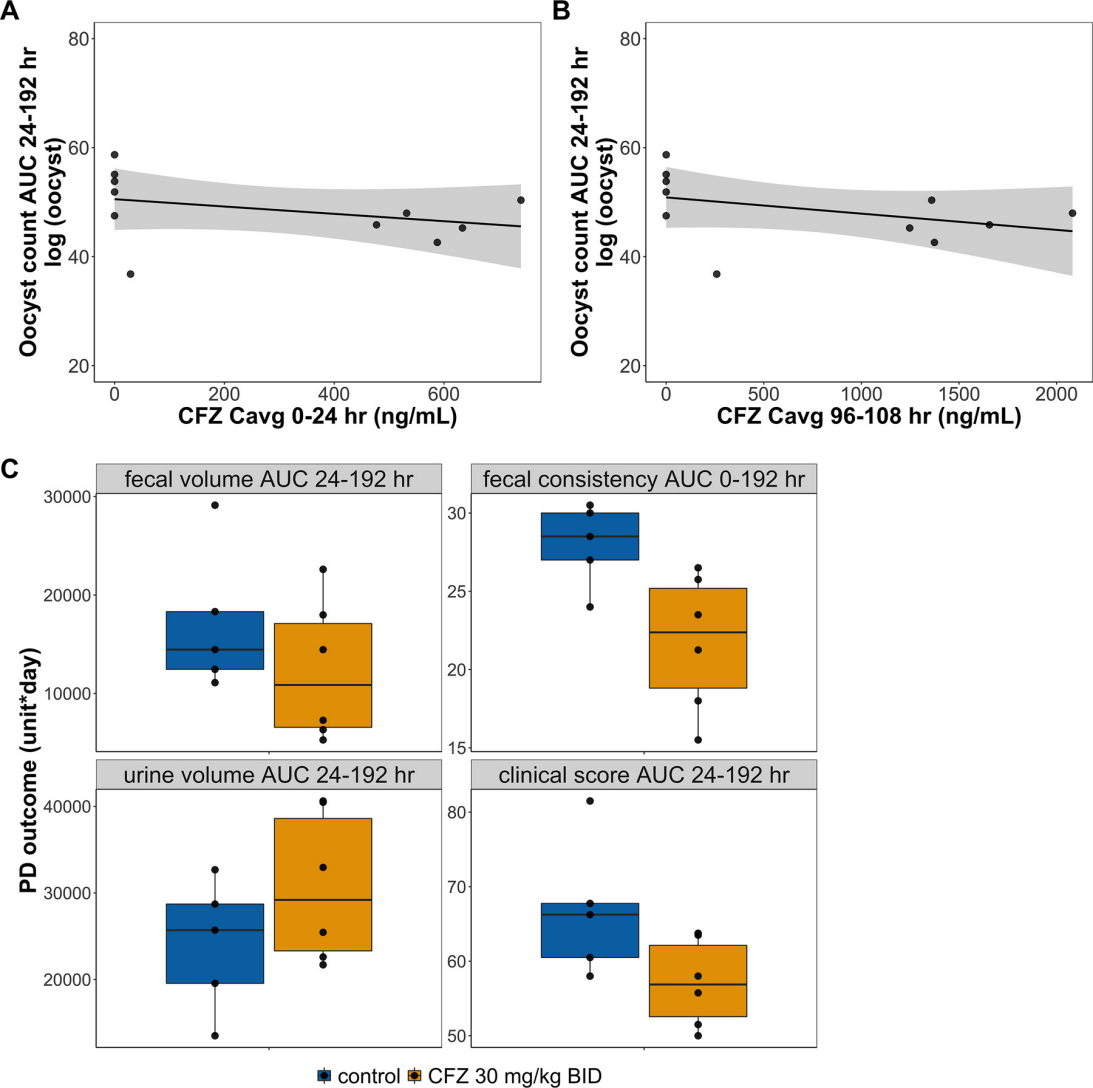
Relationship between clofazimine treatment and pharmacodynamic outcomes in calves. The oocyst count area under the curve (AUC) for the duration of the study was calculated for each calf. The association between oocyst count AUC_24–192_ and average clofazimine concentrations, *C*avg_0–24_ (A) and *C*avg_96–108_ (B), were investigated using a simple linear regression model, and the results are summarized in Table 2. Clinical outcome AUCs were also calculated for the duration of the study and compared between the treatment and control groups (C). The differences between the groups were not statistically significant after Bonferroni correction adjusting for multiple comparison. The top and bottom bars of the boxes represent the 75th and 25th percentiles, respectively, and the center lines mark the medians. The whiskers extend to the most extreme data point that is within 1.5 times the IQR away from the box. Points that fall beyond the end of the whiskers are outliers as defined by the 1.5 IQR rule.

To better evaluate the effect of clofazimine quantitatively, additional modeling approaches were undertaken. Linear regression models were employed to examine the relationship between PD outcomes, including oocyst count area under the curve from 24 to 192 h (AUC_24–192_), fecal volume AUC_24–192_, fecal consistency score AUC_0–192_, urine volume AUC_24–192_, and clinical evaluation score AUC_24–192_, and the exposure variables clofazimine *C*avg_0–24_ and clofazimine *C*avg_96–108_. The results are summarized in Table 2 and Fig. 3A and B. While clofazimine *C*avg_0–24_ was significantly and inversely associated with oocyst count AUC_24–192_ (*P* ≤ 0.01), clofazimine *C*avg_96–108_ was not significantly associated with oocyst count AUC_24–192_ (*P* = 0.24). There was a significant, negative correlation between fecal consistency score and clofazimine *C*avg_0–24_ and *C*avg_96–108_ (*P* ≤ 0.05). The clinical evaluation score AUC_24–192_ and clofazimine *C*avg_96–108_ was also negatively correlated and statistically significant (*P* ≤ 0.05). However, while fecal volume AUC_24–192_ and urine volume AUC_24–192_ may be negatively and positively correlated with clofazimine average concentrations AUC_0–192_, respectively, statistical significance was not achieved with the data collected in this experiment. Last, for easy visual comparison, box plots were used to summarize the four additional clinical endpoints by treatment groups (Fig. 3C). Although the clofazimine treatment group appears to have better outcomes for all four PD endpoints, the difference was not statistically significant after Bonferroni correction (Fig. 3C).

**TABLE 2:** Calf study linear regression model estimates

Outcome variable	Mean clofazimine concn (ng/mL)	Estimate	*P* ^*a*^	*R* ^2^
Oocyst count AUC_24–192_	*C*avg_0–24_	−0.00603	0.00570*	0.101
	*C*avg_96–108_	−0.00267	0.243	0.134
Fecal vol AUC_24–192_	*C*avg_0–24_	−8.072	0.292	0.122
	*C*avg_96–108_	−4.60	0.103	0.268
Fecal consistency score AUC_0–192_	*C*avg_0–24_	−0.00966	0.0359*	0.403
	*C*avg_96–108_	−0.00481	0.00203*	0.671
Urine vol AUC_24–192_	*C*avg_0–24_	10.80	0.224	0.160
	*C*avg_96–108_	5.73	0.0798	0.302
Clinical evaluation score AUC_24–192_	*C*avg_0–24_	−0.154	0.0762	0.308
	*C*avg_96–108_	−0.00722	0.0228*	0.455

a *P* values that reached statistical significance defined by an α value of ≤0.05 are marked with asterisks.

### Clofazimine phase 2a trial pharmacokinetics and efficacy.

**FIG 4:**
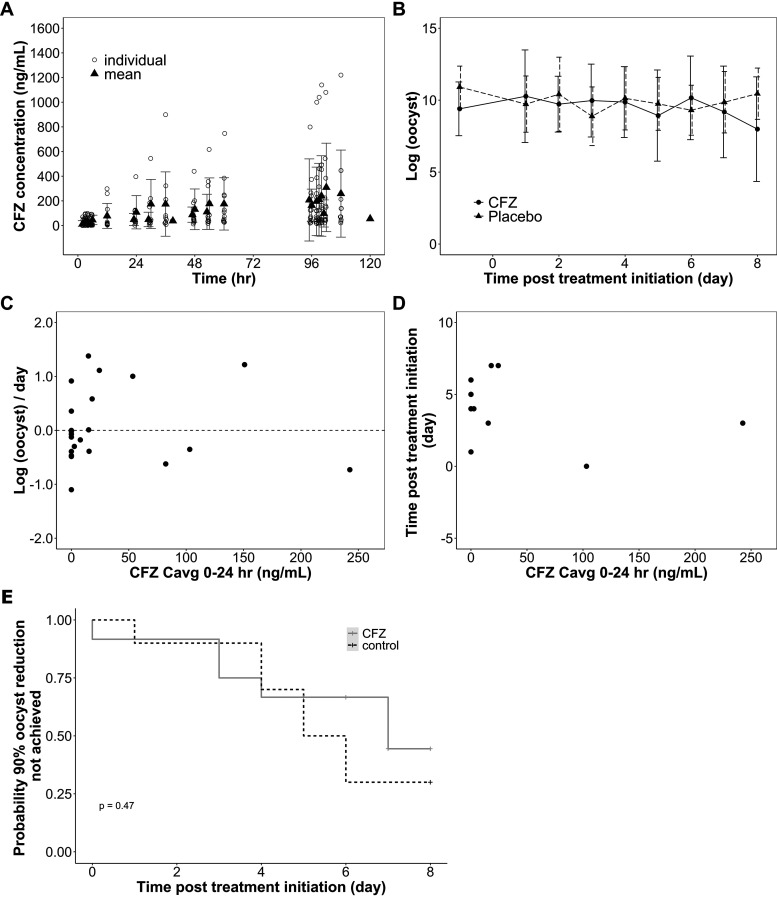
Clofazimine pharmacokinetics and pharmacodynamics in phase 2a clinical trial. HIV-infected adults with cryptosporidiosis were administered clofazimine with a dosing regimen of 100 mg 3 times daily for participants who weighed ≥50 kg (*n* = 2 participants) or 50 mg 3 times daily for participants who weighed <50 kg (*n* = 10 participants) for a duration of 5 days. (A) Mean and individual clofazimine concentrations plotted over time. (B) Stool samples were collected and assessed three times a day, and oocyst shedding was quantified using qPCR. Mean daily oocyst shedding in the first stool was compared between clofazimine treatment group and the control group. (C) For each participant, the rate of reduction in daily oocyst shedding was calculated for the first 3 days of clofazimine treatment, with the result plotted against observed clofazimine *C*avg_0–24_ to reveal a potential correlation between oocyst excretion rates and clofazimine concentrations. The dashed line marks no change in oocyst shedding rate. No correlation was clearly seen comparing the rate of reduction in oocyst shedding rates to clofazimine *C*avg_0–24_. (D) A time-to-event approach was attempted, where the time to reach 90% oocyst reduction was compared to clofazimine *C*avg_0–24_ and a significant correlation was not identified. (E) A Kaplan-Meier survival curve was used to compare the probability of achieving 90% reduction in oocyst count compared to the baseline count between clofazimine treatment group and the control group. A success was defined by having less than 10% of baseline oocyst count, and no significant difference was found, as shown by the log rank test (*P* = 0.47).

As previously reported, a phase 2a trial demonstrated that clofazimine treatment failed to improve cryptosporidiosis microbiological or clinical outcomes in a population of HIV infected adults (24). A high level of variability was observed in clofazimine concentrations among the 12 subjects who were randomized to receive treatment (Fig. 4A). Of the 12 participants, 10 weighed less than 50 kg at study enrollment and thus received 50 mg clofazimine three times a day (TID), whereas only 2 weighed over 50 kg and received 100 mg clofazimine TID. Therefore, clofazimine *C*avg_0–24_ and *C*avg_96–108_ were calculated from 10 participants and 2 participants for 50-mg-TID and 100-mg-TID doses, respectively (Table 3). Due to the high level of observed variability in pharmacokinetics and the limited number of subjects available, especially for the 100-mg-TID clofazimine dosing regimen, there was considerable uncertainty in the mean estimates. Furthermore, one participant receiving 50 mg TID clofazimine treatment had relatively high levels of clofazimine in their plasma (Fig. 4A). The extreme levels of clofazimine observed with the participant further contributed to the uncertainties in mean clofazimine *C*avg estimates. The *C*avg_0–24_ ± SD for the 10 subjects receiving 50 mg of clofazimine TID was 63.0 ± 79.4 ng/ml, and the *C*avg_0–24_ ± SD for the 2 subjects receiving 100 mg of clofazimine TID was 50.1 ± 45.3 ng/ml (Table 3). *C*avg_96–108_ ± SD was estimated to be 233 ± 340 ng/ml and 182.0 ± 60.0 ng/ml for the 50-mg-TID and 100-mg-TID groups, respectively (Table 3).

**TABLE 3:** Observed clofazimine concentrations in calf and human studies compared to estimated target exposure levels based on EC_50_s and EC_90_s established in mice

Study and dosage	*C*avg_0–24_			*C*avg_96–108_		
	Mean (SD) (ng/mL)	Observed/EC_50_	Observed/EC_90_	Mean (SD) (ng/mL)	Observed/EC_50_	Observed/EC_90_
Calf study (30 mg/kg BID, 5 days)	500 (248)	1.58	0.176	1330 (605)	4.21	0.468
Phase 2a study (humans)						
50 mg TID, 5 days	63.0 (79.4)	0.199	0.0222	233 (340)	0.737	0.0820
100 mg TID, 5 days	50.1 (45.3)	0.159	0.0176	182 (60.0)	0.576	0.0641

To compare daily oocyst shedding between treatment and placebo groups, the mean and SD of daily oocyst shedding counts in the first stool collected in the morning were determined and plotted for the two groups (Fig. 4B). No apparent effect of clofazimine treatment on lowering oocyst shedding counts was observed. Similarly, no correlation was clearly seen when the rate of reduction in oocyst shedding counts was compared to clofazimine *C*avg_0–24_ (Fig. 4C) or *C*avg_96–108_ (see the supplemental material). A time-to-event approach was also taken to explore potential clofazimine treatment effects. Defining 90% oocyst shedding count reduction as the success event, the amount of time taken to achieve success was plotted against clofazimine *C*avg_0–24_ (Fig. 4D) or *C*avg_96–108_ (see the supplemental material). No significant correlation between the concentration and response variables was detected (Table 4). Although the trend between clofazimine exposure and time to success appears to be negative, where increasing clofazimine seems to be associated with less time to achieve 90% reduction in oocyst shedding counts, it is mostly driven by two subjects with high clofazimine exposure. The lack of varying clofazimine doses and large exposure range is a great limitation of the analyses. A Kaplan-Meier curve was used as an alternative approach to detect potential differences in the amount of time taken to achieve 90% oocyst shedding count reduction between treatment and placebo groups (Fig. 4E). However, no significant difference was found, as shown by the log rank test (*P* = 0.47).

**TABLE 4:** Clinical trial linear regression model estimates

Outcome variable	Clofazimine exposure (ng/mL)	Estimate	*P*	*R* ^2^
Time to >90% oocyst reduction	*C*avg_0–24_	−0.0110	0.228	0.129
	*C*avg_96–108_	−0.00227	0.278	0.106
Rate of reduction in daily oocyst counts	*C*avg_0–24_	−0.00243	0.377	0.0715
	*C*avg_96–108_	−0.000576	0.358	0.0773

## DISCUSSION

Despite the observed efficacy in preclinical models of cryptosporidiosis, clofazimine administration failed to reduce the parasitic excretion in cryptosporidiosis in a phase 2a trial (24). Our PK/PD modeling approach described in this paper addresses the critical question of whether subtherapeutic clofazimine concentrations for cryptosporidiosis therapy contributed to the lack of clinical and microbiological efficacy against cryptosporidiosis.

Through oocyst shedding profiles observed in mice after clofazimine treatment, there was a clear dose-dependent decline in oocyst shedding within 2 days of clofazimine oral administration (Fig. 1A). The mouse model of *Cryptosporidium* infection used in our studies does not exhibit clinical signs of cryptosporidiosis (e.g., loose stool), so outcomes were limited to microbiological responses to treatment. Oocyst shedding decreased over time for all dose groups, but the magnitude and onset of the decrease in infection were dependent on the dose, with doses of ≥3 mg/kg required for an oocyst reduction rate greater than that of the control. While this promising result supported a dose-response relationship for clofazimine treatment of *Cryptosporidium* infection in mice, ideally the response to clofazimine treatment will also be associated with therapeutic concentrations (i.e., exposure-response relationship), which can provide further guidance for dose selection in studies in larger animals and clinical trials.

Since clofazimine was observed to have nonlinear pharmacokinetics at the doses used in the mouse efficacy study, the relationship between oocyst reduction and clofazimine concentrations was explored with several models. The model estimates suggested that a simple *E*_max_ model was adequate in describing the observed data, as the *E*_max_ model line captured well the shape of the observed trend (equation 1; Fig. 1C). Based on the *E*_max_ model, the estimated clofazimine concentrations that are associated with achieving 50% and 90% of the maximum rate of reduction (EC_50_ and EC_90_) were estimated to be 316 ng/ml and 2,770 ng/ml, respectively.

In addition to the rate of oocyst reduction model, a second approach taken was to use a dichotomous variable to define an outcome as either treatment success or failure for the mouse data (equation 2). Here, the success threshold of reaching a parasite reduction by at least 90% of baseline has often been used to define parasitological success in other parasitic infectious disease studies, such as clinical studies investigating malaria treatments (25, 26). The assumption for this definition of “success” was that a 90% or greater reduction in oocyst shedding 2 days after clofazimine treatment was the threshold for clinically significant treatment success, whereas anything below 90% was insufficient. The logistic model developed to characterize the relationship between the dichotomized outcomes (success/no success) and clofazimine *C*avg_0–24_ again illustrated that there was a clofazimine concentration-dependent increase in the probability of achieving success (Fig. 1D). Furthermore, the predicted EC_50_ was 394 ng/ml, which was well aligned with the EC_50_ predicted with the rate of oocyst reduction model (equation 1). It should be noted the artificially introduced threshold of 90% and the inflexible nature of using a dichotomous outcome variable are two limitations of this approach. This dichotomous outcome and logistic model approach does not distinguish between various degrees of “failure”; a case with 89% reduction in oocyst shedding was treated the same as one in which there was an entire absence of reduction, or even a case where there was an increase in oocyst shedding. This also partially explains why the logistic model-predicted EC_90_ of 589 ng/ml was much lower than the *E*_max_ model-predicted EC_90_ of 2,770 ng/ml described previously for the rate of oocyst reduction model. The black-and-white nature of the outcome variable definition led to a sharp increase in *P*, the probability of achieving success. However, these limitations do not take away the virtue of this approach. Importantly, this approach provided a second layer of easily visualizable and appreciable evidence that increasing clofazimine plasma concentration was correlated with increasing probability of achieving successful reduction in oocyst shedding within 2 days posttreatment.

Last, a time-to-event outcome variable was employed to examine the mouse data (equation 3). This approach recognizes that *Cryptosporidium* infection can be self-limiting and that oocyst numbers may drop over time even without treatment. The clearance of *Cryptosporidium* infection by the natural immune response was addressed by E_0_, which specifies that, on average, 6.50 days are needed to achieve 90% oocyst reduction without any clofazimine treatment. The treatment effect of clofazimine evaluated was its ability to induce and accelerate the elimination of oocysts in the feces. The *E*_max_ model fit to the time-to-event outcome variable and clofazimine *C*avg_0–24_ describes how increasing clofazimine exposure decreased time to clear the infection by 90% until it reached the sum of E_0_ and *E*_max_, which is the least number of days needed to achieve success and set to 1 day (Fig. 1F). From this *E*_max_ model, a clofazimine *C*avg_0–24_ of 227 ng/ml was needed to reduce the time to success by 50% of the maximum reduction possible and a *C*avg_0–24_ of 2,040 ng/ml is needed to reduce the time by 90%. This time-to-event approach further supports clofazimine treatment effect on facilitating *Cryptosporidium* oocyst clearance. However, it should be noted that this approach has its limitations. Because one mouse was excluded from the analysis, as it never reached 90% reduction during the study, the EC_50_ and EC_90_ calculated were conservative estimates, meaning that they were lower than the real values had that mouse been followed until success was achieved. Sensitivity analysis (not shown) was performed to ensure that the exclusion of the one mouse did not significantly change the EC_50_ and EC_90_ or impact our conclusion. Furthermore, as the estimates were conservative, they would not compromise the validity of our analysis. While this model is not perfect, it is adequate and effective for our use as a piece of supporting evidence.

In addition to the mouse studies, an established calf model of cryptosporidiosis was used to further investigate the *in vivo* efficacy of clofazimine. Unlike mice, the calf model exhibits clinical symptoms of cryptosporidiosis which allowed us to explore whether clofazimine treatment was associated with both microbiological and clinical outcomes. Daily oocyst count profiles of the control and the treatment group suggested that calves in the clofazimine treatment group had lower infection burden for the duration of the study (Fig. 2B). While fecal volume did not differ much between the two groups (Fig. 2C), fecal consistency score tended to be lower for the treatment group, suggesting that the treated calves produced more solid stool and had lower diarrhea severity (Fig. 2D). The urine volume was generally greater and clinical evaluation score lower for the treatment calves, indicating that treated calves were less dehydrated and had better overall health than the control calves (Fig. 2E and F). However, most PD measurements except for fecal consistency score were not collected the day of clofazimine treatment initiation but instead starting 1 day after treatment initiation. Thus, baseline pretreatment values were missing for these endpoints, and the rate of reduction in oocyst counts and percentage reduction compared to baseline values could not be calculated in a manner analogous to the mouse efficacy study. Instead, an AUC was calculated for each outcome measure using all available data. The assumption for comparing the outcome AUCs between the treatment and control groups was that these two groups were not inherently different at baseline in disease severity and parasite burden. Although there is no definitive evidence to eliminate all doubt around this assumption without baseline measurements, these calves were infected with the same number of purified disinfected C. parvum oocysts at the same age and treated in the same fashion for the duration of the study. There is no reason to believe that the two groups would be different from each other. Furthermore, fecal consistency scores, the only PD outcome that was collected at treatment initiation, were very similar between the treatment and the control groups on day 2 postinfection. This supports the notion that the treatment and control calves were similar in disease severity prior to clofazimine treatment and that differences observed between the groups are most likely due to clofazimine treatment.

Using clofazimine pharmacokinetic data collected in tandem with efficacy data for each calf, linear regression models were used to identify relationships between clofazimine concentrations and microbiological and clinical outcomes. For this approach, potential correlations were investigated between the clofazimine concentration variables *C*avg_0–24_ and/or *C*avg_96–108_ and PD outcomes, including oocyst count, fecal volume, fecal consistency score, urine volume, and clinical evaluation scores (27). A significant association between clofazimine *C*avg_0–24_ and oocyst count was identified (*P* ≤ 0.01) where an increasing average clofazimine concentration was associated with reduced oocyst counts (Table 2). In addition, a significant, correlation was also found between fecal consistency score (improved) and average clofazimine concentrations (*P* ≤ 0.05) (Table 2). Last, there was a statistically significant association between clinical evaluation score and clofazimine *C*avg_96–108_ (*P* ≤ 0.05), indicating that increasing average clofazimine concentrations were correlated with reduced infection burden, less severe diarrhea, and better overall health (Table 2). When box plots were used to compare the clinical outcomes by treatment groups, the clofazimine treated group tended to have lower fecal volume, better fecal consistency, higher urine volume, and lower clinical scores, all in favor of clofazimine treatment effect in alleviating diarrhea symptoms and improving overall health. However, the differences between the groups were not statistically significant when Bonferroni correction adjusting for multiple comparisons was used (Fig. 3C). Overall, the calf study suggested that clofazimine treatment at 30 mg/kg BID might have had some effect in reducing oocyst burden and improving clinical outcomes in calves, but its effect was at best marginal and did not produce consistent statistical significance. However, this lack of significance is likely attributable to a number of factors, including suboptimal clofazimine exposure, limited range in clofazimine exposure due to testing only one dose regimen, and limited number of calves included in this study. Using a model predicted EC_90_ of 2,770 ng/ml from the *E*_max_ oocyst reduction rate model generated with mouse data, it was calculated that the observed clofazimine *C*avg_0–24_ of 500 ng/ml and *C*avg_96–108_ of 1,330 ng/ml in calves were only 17.6% and 46.8% of the estimated EC_90_, respectively (Table 3). It is likely that doses higher than 30 mg/kg would be needed to produce more pronounced treatment effects and/or that more calves would be needed to increase the power of the study. The lack of consistently significant treatment effects in the calf study does not prove that there was no clofazimine treatment effect. Instead, this result might have been the product of suboptimal clofazimine exposure and the study being underpowered due to feasibility concerns inherent in using an outbred population of animals.

When clinical trial data of clofazimine pharmacokinetics in HIV infected adults with cryptosporidiosis were characterized here, clofazimine concentrations were clearly lower than those associated with efficacy in the preclinical disease models. When the clofazimine efficacy and pharmacokinetic data observed in the clinical trial were reported previously, there were no data available at the time to determine whether clofazimine concentrations observed in the trial were analogous to those associated with efficacy in preclinical animal models (24). Furthermore, given that the dosing regimen for the phase 2a trial was beyond the recommended dosage for mycobacterial therapy, there were limited clinical data to support clofazimine plasma levels expected in healthy individuals with the dosing regimen and no data to support plasma concentrations expected in diarrheic humans. With the preclinical data presented in this paper, it is now abundantly clear that clofazimine levels in the phase 2a trial were well below those associated with efficacy in preclinical models (Fig. 4A; Table 3). Thus, it is not surprising that the oocyst shedding profiles were very similar between the treatment and control group participants (Fig. 4B). When the rate of reduction in oocyst shedding was calculated for the clinical data just as it was done for the mouse efficacy study, there was no trend between the rates of reduction and average clofazimine concentration on day 1 of treatment (Fig. 4C). Furthermore, linear regression models and a time-to-event approach did not identify any significant associations between microbiological responses and average clofazimine concentrations on days 1 or 5 of treatment (Table 4). A Kaplan-Meier curve was used to compare the number of days taken after clofazimine treatment initiation to achieve 90% oocyst reduction between the treatment and the control groups (Fig. 4E), and clofazimine treatment did not affect the probability of achieving 90% oocyst reduction.

Although this complete lack of clinical efficacy may appear to conflict with the promising preclinical *in vivo* model results, it is explainable once observed clofazimine exposure levels in the trial are compared to the clofazimine concentrations associated with efficacy in preclinical models (Table 3). The observed clofazimine *C*avg_0–24_ and *C*avg_96–108_ in the phase 2a trial participants were more than 80% below the estimated EC_90_. Therefore, it is likely that the clofazimine doses administered in the clinical trial, which were greater than the highest approved doses to treat leprosy, were too low to treat cryptosporidiosis. The model prediction and actual observation comparisons in humans were made using the rate of oocyst reduction *E*_max_ model (equation 1) generated with mouse data, because this model rests on the smallest number of assumptions and does not involve an artificially defined dichotomous variable. Using the other two models as references would not change our conclusion, as the observed average clofazimine concentrations are still orders of magnitude below the model predicted EC_90_s. It should be noted that clofazimine has >99% plasma protein binding in mice and humans, and we did not consider the impact of plasma protein binding in our modeling approach (28, 29). Interestingly, the predicted, unbound EC_50_s for the three PK/PD models developed with mouse data were ∼3 ng/ml, which is very similar to the clofazimine EC_50_ of 7 ng/ml observed in an established *in vitro* HCT-8 coculture model of infection (21).

Finally, it should be noted that the PK/PD relationships described in this paper are informed by plasma drug levels that may not represent therapeutic concentrations at the site of action (i.e., gastrointestinal tract). Our previous studies have established that gastrointestinal therapeutic levels, not plasma levels, were associated with the efficacy of bumped kinase inhibitors for treatment of *Cryptosporidium* infection in mice (30). For clofazimine, metabolism and/or transport is not expected to impact therapeutic absorption from the gastrointestinal tract, which would be modulated by clofazimine’s solubility and permeability. Therefore, clofazimine observed in plasma would reflect compound that went into solution in the intestinal lumen and likely transited through the gastrointestinal epithelium by a transcellular pathway. Transcellular transit is important given the localization of *Cryptosporidium* spp. to intracellular but extracytosolic parasitophorous vacuoles located beneath the apical plasma membrane of infected intestinal epithelial cells, as this localization suggests that therapeutic exposure within intestinal epithelial cells is essential for therapeutic efficacy (30–33).

Moving forward, the results described here demonstrate clofazimine’s failure to treat cryptosporidiosis in a phase 2a trial was most likely due to the concentrations observed in the study participants being too low for treatment of cryptosporidiosis. The dosage of clofazimine administered in the phase 2a trial was the maximum given for leprosy in clinical practice, and an ∼70-fold-higher dose would be required to achieve an average clofazimine concentration value equal to the EC_90_ determined in mice (34). Not only would such a large dose increase present safety concerns, but also, clofazimine has very poor solubility, and it should not be assumed that increasing the dose of Lamprene, a gelatin capsule with a microcrystalline clofazimine suspension in an oil-wax base, will generate therapeutic levels in humans associated with efficacy against infection in preclinical *in vivo* models. To address challenges in improving clofazimine absorption for treatment of cryptosporidiosis, clofazimine nanoparticle powder formulations have been developed, but it is not clear whether these novel formulations will generate clofazimine concentrations required for efficacy in humans (35). In conclusion, unless alternative, safe clofazimine formulations with improved oral absorption are developed, it is unlikely that clofazimine will provide a remedy for the large number of cryptosporidiosis patients currently without a viable treatment option.

## MATERIALS AND METHODS

### Ethics statement.

All *in vivo* studies were carried out in strict accordance with the recommendations in the *Guide for the Care and Use of Laboratory Animals* of the National Institutes of Health (36). For the mouse model studies, the protocol (S13013) was approved by the Institutional Animal Care and Use Committee of the University of California, San Diego, CA (Animal Welfare Assurance number A3033-01). For the calf model study, the protocol (09-120) was approved by the Institutional Animal Care and Use Committee of the University of Arizona, Tucson, AZ (Animal Welfare Assurance number A-3248-01). Calf studies were performed in compliance with guidelines in the Animal Welfare Act and *Guide for the Care and Use of Agricultural Animals in Research and Teaching* (37). The animal biosafety level 2 (ABSL-2) facilities used were fully accredited by the American Association for Laboratory Animal Care. All efforts were made to minimize suffering of animals employed in these studies.

### Clofazimine LC-MS/MS analysis.

Clofazimine levels in mouse and calf plasma were determined by liquid chromatography with tandem mass spectrometry (LC-MS/MS) using an Agilent 1100 (Agilent, Santa Clara, CA) coupled with an AB Sciex API4000 (AB Sciex, Foster City, CA). A calibration curve was generated in plasma of the study species. Calibration curves were generated with concentrations ranging from 0.3 to 5,000 ng/mL. Two hundred fifty microliters of ice-cold acetonitrile was added to 20 μL of calibration curve sample or pharmacokinetic sample. Samples were shaken for 10 min, followed by centrifugation at 4,000 rpm for 20 min. Two hundred microliters of supernatant was removed from each sample into a new plate, and 50 μL 0.1% formic acid in high-performance liquid chromatography (HPLC)-grade water was added and mixed. Ten microliters of sample was injected for LC-MS/MS analysis. A 50- by 2.0-mm Kinetex RP 5.0-μm C_18_ column (Phenomenex, Torrance, CA) was used for analyte separation with mobile phases A (water with 0.1% formic acid) and B (acetonitrile with 0.1% formic acid) at a flow rate of 500 μL/min. The binary gradient was as follows: 10% B for 2 min, 10% B to 95% B for 2.0 to 3.5 min, 95% B for 3.5 to 5.5 min, 95% B to 10% B for 5.0 to 5.5 min, and 95% B for 5.50 to 7.0 min. Clofazimine was quantified using electrospray ionization in positive ion mode with the 473.2-to-431.0 *m/z* transition. The source temperature was set at 450°C, curtain gas at 25, ion spray voltage at 3,000, CAD gas at medium, entrance potential at 10, declustering potential at 121, and collision energy at 51. For each species, samples were analyzed over the course of a single day, and the coefficient of variation for calibration curve samples were all <15% with the exception of one concentration (5,000 ng/mL in mouse plasma had a 23% coefficient of variation). For both the mouse and calf calibration curves, the *R*^2^ values were >0.95. The limit of detection (LOD) and lower limit of quantification (LLOQ) for clofazimine in mouse plasma were 0.15 and 0.3 ng/mL, respectively. For the calf study, the LOD and LLOQ were 0.3 and 1.2 ng/mL, respectively. The human clinical trial samples were analyzed by Q2 solutions as previously described using an LC-MS/MS assay developed and validated according to FDA guidelines (24).

### Mouse pharmacokinetic study.

Eight-week-old male C57BL/6 mice were used to characterize the pharmacokinetics of clofazimine after a single oral dose. Clofazimine (USP reference standard, Sigma-Aldrich) doses of 0.03, 0.1, 0.3, 1, 3, 10, 30, 100, and 300 mg/kg were formulated in 0.5% methylcellulose and 0.5% Tween-80 (MC-Tween) and dosed at 5 mL/kg. Each dose group contained 6 mice randomized to 2 equal cohorts, where blood samples were collected at 0.5, 3, 8, and 120 h postdose for cohort 1 and at 1, 8, 24, and 240 h postdose for cohort 2.

### Mouse efficacy study.

The mouse model used to characterize clofazimine pharmacodynamics has been described in detail previously (21). Briefly, four- to five-week-old female C57BL/6 gamma interferon-deficient (IFN-γ^−/−^) mice were inoculated with 10^6^ purified C. parvum oocysts (Iowa strain) at a density of 5 × 10^6^ oocysts/mL in sterile water via oral gavage. Four days postinfection, clofazimine was administered as a single oral dose to infected mice at 0.03, 0.1, 0.3, 1, 3, 10, 30, 100, and 300 mg/kg. Each dose group contained four male mice. Mice were placed in isolation to allow collection of feces at 24-h intervals posttreatment for each mouse. Fecal pellets were weighed immediately after collection, placed in 0.5 mL 2.5% potassium dichromate solution, and stored at 4°C until processing. Oocysts in fecal samples were quantified using a Guava EasyCyte flow cytometer and CytoSoft data acquisition and analysis software (v5.3; Guava Technologies, Inc.).

### Calf pharmacokinetic and efficacy study.

All calves were obtained from the same United States Department of Agriculture (USDA)-licensed closed-herd dairy vendor. Calves were fed commercial colostrum replacer within 2 h after birth (bovine IgG colostrum replacement; Land O’Lakes, Shoreview, MN) per label instructions. A total of 12 calves were used, with 6 randomly assigned by the Microsoft Excel random number generation tool (Redmond, WA) to the treatment group and 6 to the control group. Experiment personnel were blind to the treatment and control assignments during the course of the study. All calves were housed in an ABSL-2 facility in separate containment rooms. Precautions and disinfection measures were taken for the deliveries and housing of these calves to prevent unintended *Cryptosporidium* or other enteropathogen infection. The calves were fed antibiotic-free milk replacer (Nutrena Snowflakes calf milk II-Utiliz milk replacer; Cargill Animal Nutrition, Minneapolis, MN) twice daily from 12 h of age until termination of the experiment at day 10 postinfection. An oral electrolyte solution (Re-Sorb; Pfizer) was supplemented once diarrhea developed in an animal.

At 36 to 48 h of age (study day 0), each calf was infected by oral inoculation of 5 × 10^7^ purified disinfected C. parvum oocysts (Iowa strain) (27). Starting at study day 2 (2 days postinfection), each calf in the treatment group received 30 mg/kg clofazimine twice daily (BID) over 5 days for a total of 10 doses. Clofazimine was dosed in MC-Tween with a total dose volume of 1 mL/kg. Control calves received only MC-Tween. Over the 5-day dosing period, a total of 16 blood samples were collected to characterize clofazimine pharmacokinetics. On study days 2 and 6, pharmacokinetic samples were collected immediately prior to the first dose of the day and 1, 2, 4, 12, 13, and 24 h postdose. In addition, predose pharmacokinetic samples were collected for doses 5 and 7. Stool samples were collected every 24 h starting on study day 3. The total volume of feces excreted for successive 24-h collections was recorded. Total daily oocyst counts for each calf were determined as previously described (27). Briefly, real-time quantitative PCR was used to quantify C. parvum oocysts from feces collected over successive 24-h periods which had been well mixed using a commercial blender to ensure sample uniformity. Calves were also assigned numerical scores for the following variables twice daily: clinical symptoms, general health (willingness to rise, stance, rectal temperature, appetite and food intake, attitude, and hydration status), presence or absence of diarrhea, and fecal consistency (38). All calves with the exception of one control calf were euthanized on study day 10. The control calf euthanized on study day 9 was withdrawn from the study one day early due to bloating and severe diarrhea.

### Clofazimine phase 2A trial for treatment of cryptosporidiosis.

The study design and outcomes were described in detail previously (24). Briefly, the trial was a single-center, randomized, double-blind, placebo-controlled phase 2a trial conducted in Blantyre, Malawi (NCT number 03341767). HIV-infected adults between 18 and 65 years of age, weighing over 35.4 kg, who had received antiretrovirals for at least 1 month, experienced at least 14 days of diarrhea, and tested positive for *Cryptosporidium* infection by qPCR were eligible to enroll. Subjects were randomized 1:1 to receive oral clofazimine (*n* = 12) or placebo (*n* = 10). The dosage of clofazimine (Lamprene formulation) was the maximum given in clinical practice, 100 mg three times daily (TID) for participants who weighed ≥50 kg or 50 mg TID for participants who weighed <50 kg. Participants were dosed with Lamprene or placebo for a total of 5 days. Approximately 30 min before each clofazimine dose administration, subjects received Plumpy’Soy or Plumpy’Nut nutritional supplement.

Blood samples for pharmacokinetic measurements were collected on day 1 predose, at 2, 3, and 4 h after the first dose, and immediately prior to the second and third doses. On days 2 and 3, blood draws were taken immediately prior to each dose. On day 5, samples were taken immediately prior to each of the three doses and 2, 3, and 4 h after the first dose. Clofazimine concentration assessment was performed by Q2 Solutions (Ithaca, NY, USA) using liquid chromatography-tandem mass spectrometry (LC-MS/MS). The assay methods used were validated according to U.S. Food and Drug Administration guidelines for quantification of clofazimine within the range of 1.0 ng/mL to 1,000 ng/mL in human plasma. Stool samples were collected and assessed three times a day prior to each dose administration from day 1 to day 5 and once on day 6 prior to discharge. The presence of diarrhea and severity of diarrhea were recorded. All *Cryptosporidium* shedding was quantified using qPCR.

### Pharmacokinetic-pharmacodynamic modeling.

For all *in vivo* studies, pharmacokinetic parameters were initially estimated through noncompartmental analysis (NCA) using Phoenix WinNonlin version 8.3 (Certara, Princeton, NJ). To determine *Cryptosporidium* oocyst shedding rates for each subject, a linear regression line was fitted to log-transformed daily oocyst shedding counts for the first 3 days beginning at the day of clofazimine treatment initiation. To determine the percent reduction in oocyst shedding, the oocyst count 2 days after initial treatment was divided by the baseline oocyst count on the day of treatment initiation for each study subject, multiplied by 100, and subtracted from 100%.

To quantitatively evaluate the relationship between oocyst reduction rate and clofazimine dose in mice, an *E*_max_ model was fitted using mean clofazimine concentration for the first 24 h following treatment administration (*C*avg_0–24_) at each dose determined from the mouse pharmacokinetic study and the individual rate of oocyst reduction following clofazimine administration as calculated from the mouse efficacy study (equation 1).

(1) $$\text{RR}=\;{\text{ E}}_{0}\;+\;\frac{E_{\text{max}}\;\times \;C{\text{avg}}_{0-24}^{\text{γ}}}{{\text{EC}}_{50}^{\gamma }\;+\;C{\text{avg}}_{0-24}^{\text{γ}}}$$

where RR is the rate of reduction in daily oocyst shedding, E_0_ is the rate of reduction observed in the vehicle control group, γ is the Hill coefficient, *E*_max_ is the estimated maximum rate of reduction in daily oocyst shedding, and EC_50_ is the average clofazimine concentration at which half the maximum rate of reduction in oocyst shedding can be achieved.

A dichotomous variable was also designed to explore the relationship between treatment success and average clofazimine concentration. A success event was defined as achieving a ≥90% reduction in oocyst count by comparing day 6 oocyst shedding numbers to the baseline oocyst count at treatment initiation (day 4 postinfection). A logistic model was developed to describe the relationship between the probability of success for each individual mouse in the efficacy study and the mean clofazimine concentration *C*avg_0–24_ for its corresponding dose group in the pharmacokinetic study (equation 2).

(2) $$\log\left(\frac{P}{1\;-\;P}\right)={\text{β}}_{0}\;+\;{\text{β}}_{1}\times C{\text{avg}}_{0-24}$$

where *P* is the probability of achieving a success event (i.e., the probability of achieving a ≥90% reduction in oocyst count), EC_50_ is the average clofazimine concentration at which the probability of success is 0.50 and can be estimated by substituting 0.50 for *P* in the equation, and β_0_ and β_1_ are the intercept and slope of the equation, respectively, describing how the logit function of *p* changes in response to changes in *C*avg_0–24_.

Last, a time-to-event outcome variable was adopted to study the relationship between average clofazimine concentration and the time to achieve treatment success, defined as a ≥90% reduction in oocyst counts compared to day 4 postinfection (day of treatment initiation). The number of days following clofazimine administration each mouse took to achieve success was compared to the *C*avg_0–24_ for its corresponding dose group in the pharmacokinetic study. Since one mouse at the 0.1-mg/kg dose did not achieve success by study termination, it was excluded from this analysis (*n* = 39). An *E*_max_ model similar to equation 1 was fitted to describe the relationship (equation 3).

(3) $$\text{TTE}=\;{\text{ E}}_{0}\;+\;\frac{E_{\text{max}}\times C{\text{avg}}_{0-24}^{\text{γ}}}{{\text{EC}}_{50}^{\gamma }\;+\;C{\text{avg}}_{0-24}^{\gamma }}$$

where E_0_ is the average number of days after treatment initiation to achieve success for the control group, and this value was fixed at 6.50 days based on the observed data, *E*_max_ is the largest possible change in the number of days to achieve success due to a treatment effect and was set to −5.50 days, TTE is the time to event (achieving ≥90% oocyst reduction) in days, γ is the Hill coefficient, and EC_50_ is the *C*avg_0–24_ at which the time to success is reduced by half of the estimated maximum change possible. By fixing E_0_ and *E*_max_, the lower bound of TTE was designated as 1 day. In other words, when the clofazimine concentration is high enough to produce the maximum treatment effect, the least amount of time required to achieve treatment success would be the sum of E_0_ and *E*_max_, which is 1 day after clofazimine treatment initiation. This would be the minimum duration possible and plausible given the daily sampling schedule.

Since no baseline oocyst shedding data on study day 2 (day of clofazimine treatment initiation) were collected in the calf study, the rate of reduction in oocyst shedding immediately following treatment initiation and percent reduction comparing daily oocyst counts to the predose baseline cannot be calculated as done for the mouse study. Instead, areas under the curves (AUCs) were calculated for oocyst count, fecal volume, fecal consistency, urine volume, and clinical score for the entire duration for which each outcome was assessed. The relationship between the pharmacodynamic (PD) endpoint and clofazimine *C*avg_0–24_ and *C*avg_96–108_ was described by a linear regression model (equation 4).

(4) $$\text{PD endpoint}={\text{β}}_{0}\;+\;{\text{β}}_{1}\times C\text{avg}$$

The outcome variables (PD endpoint) examined include oocyst count AUC_24–192_ (from 24 to 192 h after clofazimine initiation), fecal volume AUC_24–192_, fecal consistency score AUC_0–192_, urine volume AUC_24–192_, and clinical evaluation score AUC_24–192_. β_0_ is the intercept of the equation and estimates the level of the PD endpoint without clofazimine treatment. β_1_ is the slope, describing the direction and magnitude with which a PD endpoint changes with changing clofazimine average concentration. The *C*avg examined include *C*avg_0–24_ and *C*avg_96–108_.

### Statistical analysis.

Linear regression, logistic regression, and *E*_max_ models were used to model the associations between outcome and clofazimine concentration variables. Kaplan-Meier curves and log-rank tests were used for time-to-event comparisons. Bonferroni correction for multiple comparisons was employed for comparing fecal volume, fecal consistency, urine volume, and clinical scores of the treatment and control groups in the calf study. Statistical analyses were all carried out using R version 3.6.1 (5 July 2019).
