# Supplementary material for: Pharmacokinetics and Pharmacodynamics of Clofazimine for Treatment of Cryptosporidiosis
Source: Antimicrob Agents Chemother. 2022 Jan 18;66(1):e01560-21. doi: 10.1128/AAC.01560-21 (PMC8765308; doi:10.1128/AAC.01560-21)

Supplemental Materials: Pharmacokinetics and pharmacodynamics of clofazimine for treatment of cryptosporidiosis

Cindy X. Zhang[a], Melissa S. Love[b], Case W. McNamara[b], Victor Chi[b], Ashley K. Woods[b], Sean Joseph[b], Deborah A. Schaefer[c], Dana P. Betzer[c], Michael W. Riggs[c], Pui-Ying Iroh Tam[d,e], Wesley C. Van Voorhis[f], Samuel L.M. Arnold[a,f#]

[a]Department of Pharmaceutics, University of Washington, Seattle, Washington, USA

[b]Calibr, a division of The Scripps Research Institute, La Jolla, California, USA

[c]School of Animal and Comparative Biomedical Sciences, College of Agriculture and Life Sciences, University of Arizona, Tucson, Arizona, USA

[d]Malawi-Liverpool Wellcome Trust Clinical Research Programme, Blantyre, Malawi.

[e]Liverpool School of Tropical Medicine, Liverpool, UK.

[f]Department of Medicine, University of Washington, Seattle, Washington, USA

Running Head: Clofazimine pharmacokinetics and pharmacodynamics

#Address correspondence to Samuel L. M. Arnold, slarnold@uw.edu.

Keywords: Cryptosporidiosis, pharmacokinetics, pharmacodynamics, PK/PD, gastrointestinal, infectious diseases

Supplemental Figure 1: Calf PD outcomes vs. clofazimine Cavg

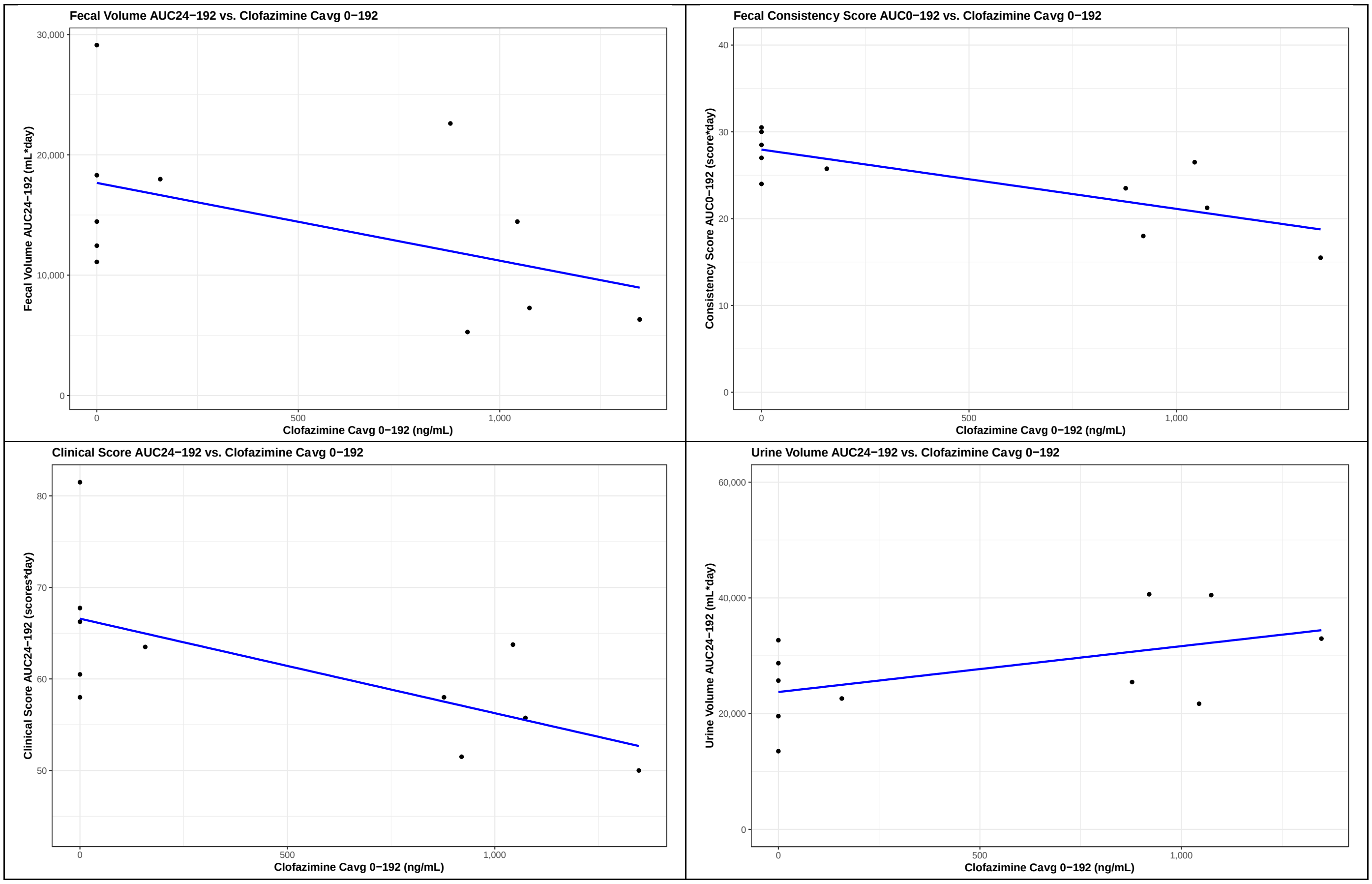

Supplemental Figure 2: Clinical Trial: rate of reduction vs. CFZ Cavg 96-108.

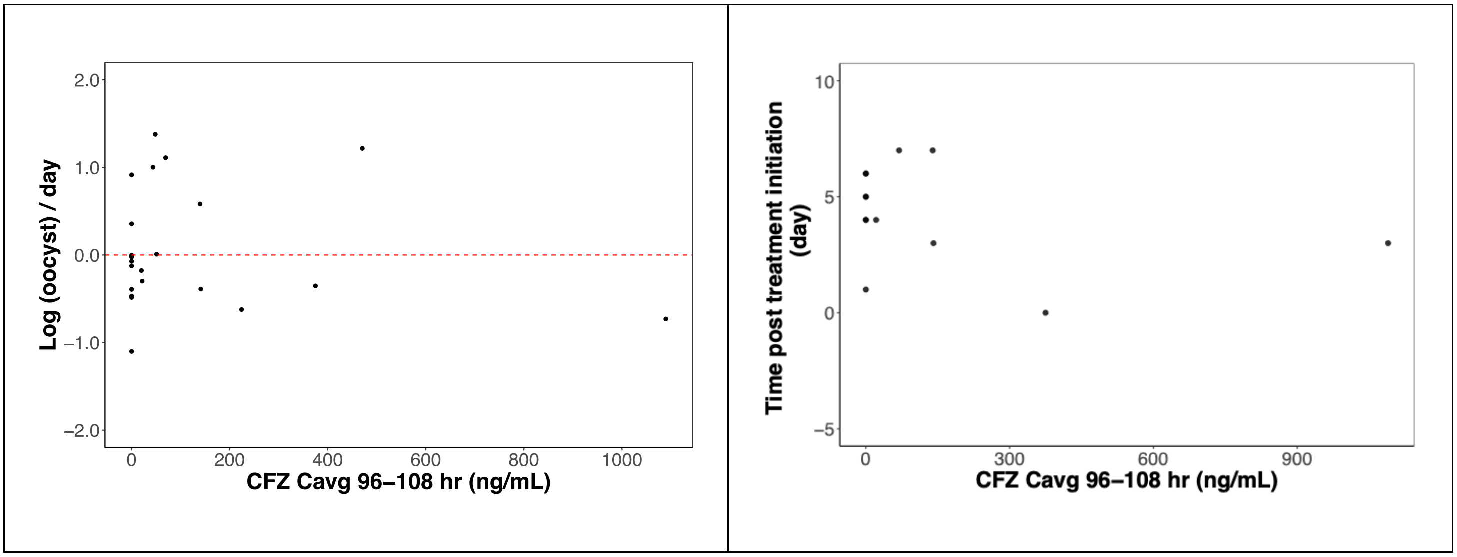

Supplement: Supplemental file 1 — Supplemental figures. Download AAC.01560-21-s0001.pdf, PDF file, 0.10 MB [file aac.01560-21-s0001.pdf]
